# Chromatin Modifications as Determinants of Muscle Stem Cell Quiescence and Chronological Aging

**DOI:** 10.1016/j.celrep.2013.05.043

**Published:** 2013-06-27

**Authors:** Ling Liu, Tom H. Cheung, Gregory W. Charville, Bernadette Marie Ceniza Hurgo, Tripp Leavitt, Johnathan Shih, Anne Brunet, Thomas A. Rando

**Affiliations:** 1 The Glenn Laboratories for the Biology of Aging and Department of Neurology and Neurological Sciences, Stanford University School of Medicine, Stanford, CA 94305, USA; 2 Department of Genetics, Stanford University School of Medicine, Stanford, CA 94305, USA; 3 Neurology Service and RR&D Center of Excellence, VA Palo Alto Health Care System, Palo Alto, CA 94304, USA

## Abstract

The ability to maintain quiescence is critical for the long-term maintenance of a functional stem cell pool. To date, the epigenetic and transcriptional characteristics of quiescent stem cells and how they change with age remain largely unknown. In this study, we explore the chromatin features of adult skeletal muscle stem cells, or satellite cells (SCs), which reside predominantly in a quiescent state in fully developed limb muscles of both young and aged mice. Using a ChIP-seq approach to obtain global epigenetic profiles of quiescent SCs (QSCs), we show that QSCs possess a permissive chromatin state in which few genes are epigenetically repressed by Polycomb group (PcG)-mediated histone 3 lysine 27 trimethylation (H3K27me3), and a large number of genes encoding regulators that specify nonmyogenic lineages are demarcated by bivalent domains at their transcription start sites (TSSs). By comparing epigenetic profiles of QSCs from young and old mice, we also provide direct evidence that, with age, epigenetic changes accumulate and may lead to a functional decline in quiescent stem cells. These findings highlight the importance of chromatin mapping in understanding unique features of stem cell identity and stem cell aging.

## Introduction

Because every cell in a metazoan (with rare exceptions; Matthews and Oettinger, 2009) possesses the same genetic information, the identity of distinct types of cells is established at the epigenetic level. The epigenome determines the pattern of gene expression that gives each cell its distinct characteristics and functions (Spivakov and Fisher, 2007). Adult stem cells have the remarkable abilities both to self-renew and to differentiate into functional progeny during tissue homeostasis and regeneration. To date, the epigenetic characteristics of stem cells that are associated with their unique features such as quiescence, self-renewal, and differentiation remain largely unexplored.

The eukaryotic genome is packaged into chromatin whose basic unit is the nucleosome. Each nucleosome is composed of four core histones: H2A, H2B, H3, and H4 (Luger et al., 1997). The amino-terminal tails of these core histones are exposed on the surface of nucleosomes and are subject to a wide range of posttranslational modifications (Kouzarides, 2007; Tan et al., 2011b). Many of these histone modifications are associated with gene activation or repression and may have a regulatory role in transcriptional initiation and elongation. For example, active promoters are generally marked by trimethylation of histone 3 lysine 4 (H3K4me3), actively transcribing genes are commonly marked by a broad H3K4me3 domain around their transcription start sites (TSSs) and trimethylation of histone 3 lysine 36 (H3K36me3) in the gene body, and Polycomb group (PcG) complex-mediated trimethylation of histone 3 lysine 27 (H3K27me3) is associated with transcriptional repression (Kouzarides, 2007). On one hand, the histone modifications dynamically change during transcription as a consequence of the recruitment of chromatin-modifying enzymes by transcription factors and the RNA polymerase (Pol) II complex (Kouzarides, 2007). On the other hand, the formation of certain combinations of histone modifications around transcription factor consensus sequences precedes, and therefore may direct, the binding of a transcription factor (Guccione et al., 2006). With a growing number of histone modifications identified in recent years (Tan et al., 2011b), the model that the combination of histone modifications comprises a complex “histone code” as a source of inheritable epigenetic information has become increasingly appealing (Strahl and Allis, 2000).

Equally interesting is the growing body of evidence that because the histone code can be transmitted from one cell generation to the next (Moazed, 2011), it can act as the cellular memory of its identity. Such cellular memory is particularly important for adult stem cells as they self-renew to maintain the stem cell pool that needs to last the lifetime of an organism. Stem cells alternate between a quiescent state and a cycling state in response to stimuli that promote tissue maintenance or repair. Following their activation, stem cells give rise to a daughter that is destined to return to quiescence, a process during which the progenitor relies on an epigenetic memory across the chromatin to restore the original transcriptional state. To decipher the histone code of quiescent stem cells is therefore essential not only to understand the identity of stem cells but also to determine whether the decline of stem cell function during aging or in specific diseases is a consequence of a compromised epigenetic memory.

Skeletal muscle is a postmitotic tissue that exhibits an extremely low turnover in the absence of disease or injury. At the same time, muscle possesses remarkable regenerative capacity mediated by satellite cells (SCs) that reside in close association with individual myofibers, underneath the fiber's basal lamina (Mauro, 1961). Consistent with the low turnover of the muscle, SCs in adult animals are mitotically quiescent (Schultz et al., 1978) and, therefore, provide an excellent model to study stem cell quiescence. As an organism grows older, the resident stem cells are exposed to a deteriorating environment and experience chronological aging. In stem cells with high turnover, the effects of chronological aging are superimposed upon the effects of the replicative aging that results from DNA replication and cell division (Liu and Rando, 2011). On the contrary, SCs experience minimal replicative aging due to their low turnover. They are thus a good model to study the consequence of chronological aging of quiescent stem cells.

In this study, we isolated quiescent and activated SCs (QSCs and ASCs, respectively) from both young and old mice and applied gene expression microarray analysis to elucidate the transcriptional profile of QSCs and how it changes when SCs activate during muscle regeneration after an acute injury. Chromatin immunoprecipitation sequencing (ChIP-seq) technology was used to acquire H3K4me3, H3K27me3, and H3K36me3 profiles in QSCs from young and aged mice and in ASCs from young mice. Our analysis of these profiles, in conjunction with the comparison to the chromatin profiles from other types of stem cells, revealed a general lack of a repressed chromatin configuration in QSCs. We further demonstrated that the chronological aging of QSCs was accompanied by the accumulation of repressed chromatin domains that may be linked to their functional decline with age.

## Results

### Isolation of QSCs and ASCs from Limb Muscles

In order to obtain a pure population of SCs for transcriptional and epigenetic analysis, we developed an isolation protocol that selectively enriched SCs (as VCAM1^+^/CD31^−^/CD45^−^/Sca1^−^ cells) from both uninjured and injured limb muscles of adult mice (Figure S1). The sensitivity and specificity of our sorting scheme were confirmed by using a mouse strain in which SCs and their progeny were genetically labeled by YFP expression (Figure S2A). FACS analysis of all mononucleated cells in the muscle of tamoxifen-treated Pax7^CreER/+^; ROSA26^eYFP/+^ mice revealed that all YFP-expressing cells were positive for VCAM1 expression and negative for CD31, CD45, and Sca1 and that YFP-expressing cells could be found only in VCAM1^+^/CD31^−^/CD45^−^/Sca1^−^ cells (Figures S2B and S2C). Moreover, we performed microarray analysis on VCAM1^+^/CD31^−^/CD45^−^/Sca1^−^ QSCs or ASCs isolated from wild-type mice and YFP-expressing cells from the Pax7^CreER/+^; ROSA26^eYFP/+^ mice. Our comparison revealed that the gene expression profiles were nearly identical in cells isolated by these two different approaches (Figures S2D-S2I).

### Gene Expression Pattern of QSCs

**Figure 1:**
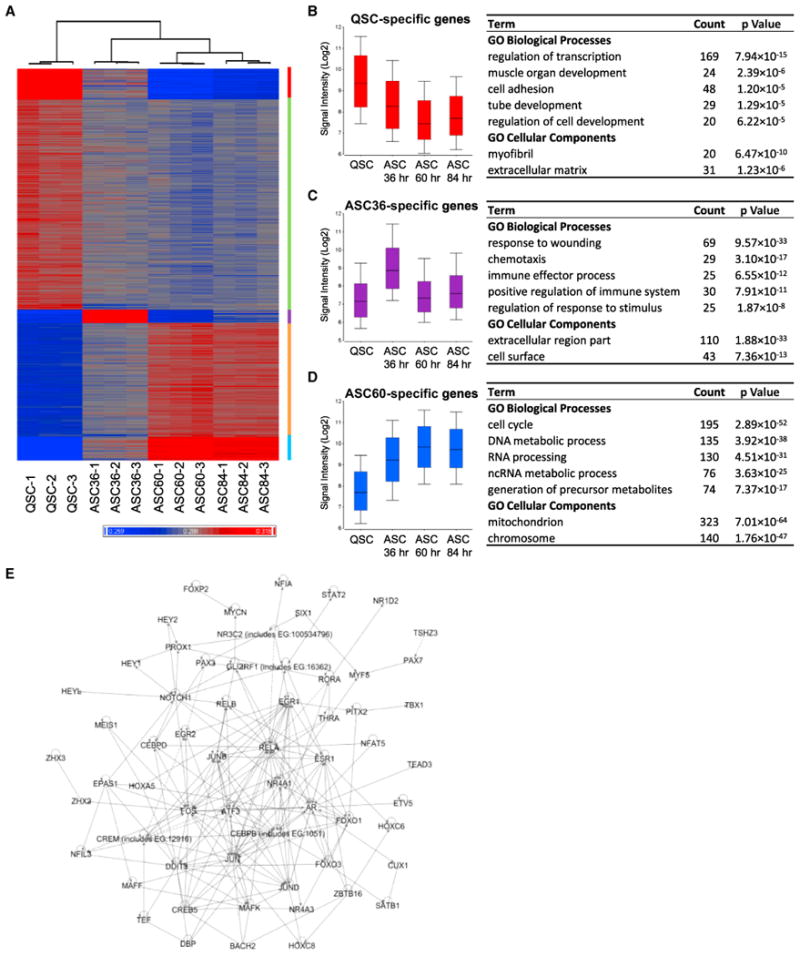
Microarray Analysis of QSCs and ASCs during Muscle Regeneration (A) Clustered heatmap of the gene expression profiles of QSCs and ASCs prior to and 36, 60, and 84 hr after muscle injury. Three replicates were used for each sample. (B–D) Box and whisker plots of the expression value (Log2 intensity) and GO analysis of genes specific to QSCs and ASCs 36 and 60 hr postinjury. (E) Transcription network in QSCs. Pathway analysis of the transcription factors that expressed at a substantially higher level in QSCs than ASCs was performed with the Ingenuity software package. Interactions were found between 59 transcription factors. In the map, transcription factors with the most interactions were placed in the center, and those with the least interactions were placed at the periphery. See also Figures S1 and S2 and Table S1.

By microarray analysis, we identified genes that were specific to QSCs, ASCs in the early phase of regeneration (36 hr after injury), and ASCs at the peak of the transit-amplifying phase (60-84 hr after injury) (Figure 1; Table S1). Gene Ontology (GO) analysis was also performed to understand the functional significance of the genes specific to QSCs and ASCs (Figures 1B-1D). Genes specific to QSCs were significantly enriched for GO terms linked to the regulation of transcription (Figure 1B). A total of 71 transcription factors expressed at a high level in QSCs. Based on known protein-protein interactions by biochemical and genetic studies, we generated a transcription network with 59 of these 71 QSC-specific transcription factors (Figure 1E). This transcription network includes known regulators of SC function such as myogenic transcriptional factors Pax7 and Myf5 and target genes of the Notch pathway (Conboy et al., 2005; Gayraud-Morel et al., 2007; McKinnell et al., 2008; Bjornson et al., 2012), as well as a number of regulators, such as Foxo1, Foxo3, Gli2, and Nfat5 (Table S1).

Local infiltration of lymphocytes and macrophages after muscle injury is essential for proper regeneration (Pelosi et al., 2007). The genes that were highly expressed in ASCs 36 hr after muscle injury were remarkably enriched for GO terms associated with wound-healing response and chemotaxis (Figure 1C), indicating that immediately following activation, SCs, in conjunction with other types of cells in the muscle, provide the chemotactic gradient that recruits immune cells to the site of injury. The gene expression profiles of ASCs isolated 60 and 84 hr postinjury are highly similar (Figure 1A). Consistent with our observation that the proliferation of SCs peaks between 2 and 4 days after muscle injury, the genes that were specific to ASCs 60 and 84 hr after injury were enriched for GO terms related to the positive regulation of the cell cycle and metabolic processes (Figure 1D), a character of highly proliferative cells.

Of note, there are genes associated with myogenic lineage progression, most notably MyoD, that do not appear in the list of genes “specific to ASCs” because in fact, their expression levels were unexpectedly high in QSCs, whereas their protein levels remained undetectable (Figures S1B and S1E). Therefore, they do not appear in either the “QSC-specific” or the “ASC-specific” lists because they are present in both. This pattern, an expression of transcript uncoupled from expression of protein, was also observed in our ASC transcriptional profiles, where we observed high transcript levels of many genes whose protein products were not detected until much later in terminal differentiation (see Figure 5C).

In summary, our microarray analysis of QSCs and ASCs reveals a gene expression signature of QSCs and regulators of SC function. The gene expression changes in ASCs, particularly during the early phase of muscle regeneration, reflect changes in the niche that can modulate SC activation and proliferation. Equally important is the availability of a steady-state gene expression profile for our subsequent analysis of chromatin marks and their dynamic changes during SC activation.

### Genome-wide Profiles of H3K4me3, H3K27me3, and H3K36me3 in QSCs and ASCs

In order to gain insight into the chromatin state of adult stem cells in quiescence and the dynamic changes of chromatin during stem cell activation, we performed ChIP-seq using QSCs and ASCs. We analyzed the enrichment of H3K4me3, H3K27me3, and H3K36me3 across the genome. Analysis of the general profiles of these ChIP-seq data sets revealed conventional distribution of these histone modifications relative to gene features in that H3K4me3 was found commonly at the TSSs of genes, and H3K36me3 was found enriched in the coding regions. The genes that were expressed at high levels in QSCs by microarray analysis were all marked by H3K4me3 and devoid of H3K27me3 at their TSSs, and they were generally marked by H3K36me3 that aligned well with the position of exons (Figure S3A).

**Figure 2:**
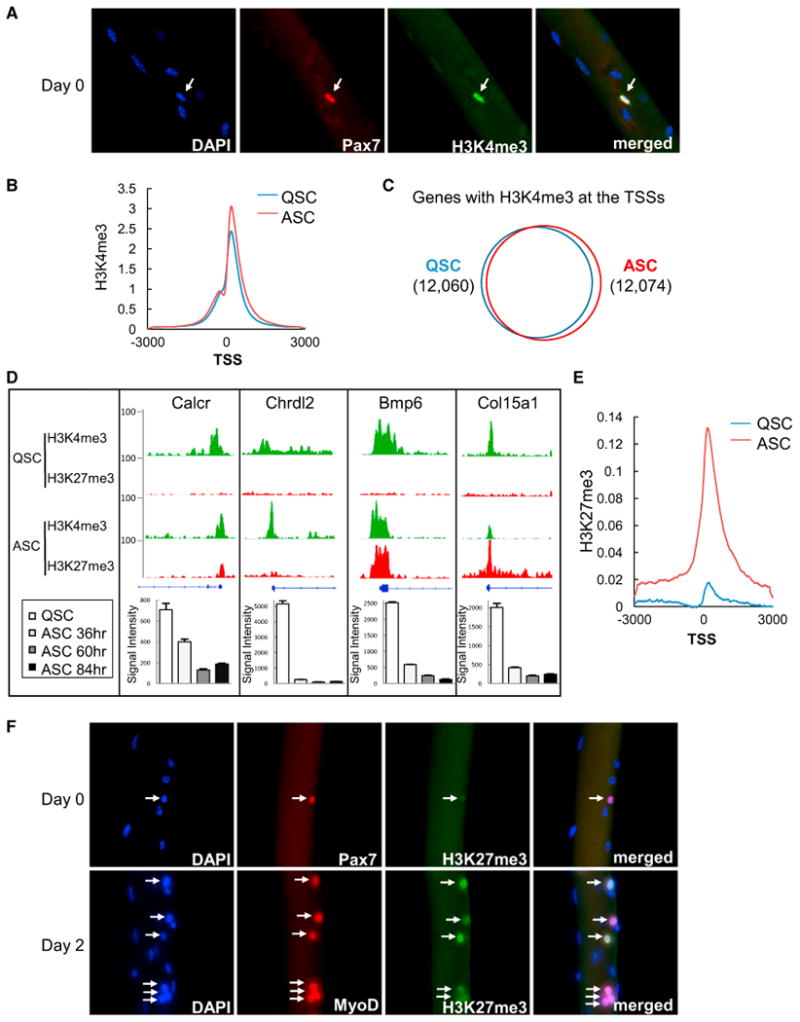
The Global Profiles of H3K4me3 and H3K27me3 in QSCs and ASCs (A) Immunofluorescence of Pax7 and H3K4me3 on freshly isolated single fibers. The arrows indicate a fiber-associated QSC that is positive for both Pax7 and H3K4me3. (B) Distribution of H3K4me3 around the TSSs in QSCs and ASCs. Normalized tag intensity of H3K4me3 3 kb upstream and downstream of the TSSs across the genome is shown in the plot. (C) Venn diagram of genes marked by H3K4me3 at their TSSs in QSCs and ASCs. (D) H3K4me3 and H3K27me3 distribution at the TSS of genes that expressed at high levels in QSCs but were downregulated in ASCs. The H3K4me3 and H3K27me3 profiles of representative genes are shown in the top panels, and the level of changes in their expression upon SC activation revealed by microarray analysis is shown in the bottom bar graphs. Error bars represent SDs. (E) Distribution of H3K27me3 around the TSSs in QSCs and ASCs. Normalized tag intensity of H3K27me3 3 kb upstream and downstream of the TSSs across the genome is shown in the plot. (F) Immunofluorescence of freshly isolated myofibers with Pax7 and H3K27me3 antibodies (top panels) and fibers cultured for 2 days ex vivo with MyoD and H3K27me3 antibodies (bottom panels). Images were acquired with the same exposure and gain. Fiber-associated SCs are indicated by the arrows, and all other DAPI^+^ nuclei are myonuclei within the fiber. See also Figures S3 and S4 and Table S2.

QSCs exhibited high levels of global H3K4me3 (Figure 2A). In QSCs, nearly 50% of annotated genes in the mouse genome, including nearly all housekeeping genes and cell-cycle genes, were marked with H3K4me3 at their TSSs (Figure S3B). Neither the number nor the identity of genes marked by H3K4me3 at their TSSs changed significantly in ASCs compared to QSCs (Figures 2B, 2C, and S3B). Consistent with the notion that H3K4me3 alone does not predict the transcriptional state of a gene but, instead, marks it for transcriptional activation (Guenther et al., 2007), genes that were transcriptionally upregulated upon SC activation, including those that were not expressed in quiescence, had already been marked by H3K4me3 in QSCs. Furthermore, QSC-specific genes retained H3K4me3 at their TSSs after their transcription was rapidly downregulated upon entering the cell cycle (Figure 2D).

In contrast to the fairly constant level of H3K4me3 across the genome and the large number of genes marked by H3K4me3 in both QSCs and ASCs, the level of H3K27me3 was low in QSCs and dramatically increased in ASCs (Figures 2E and 2F). Only 2,019 genes were marked by H3K27me3 at their TSSs in QSCs (Figure S3B; Table S2). The expression levels of these H3K27me3-marked genes were either undetectable or extremely low (Figure S4A). Upon SC activation, the genes marked by H3K27me3 at their TSSs doubled in number (Figure S3B). Many genes that were highly expressed in QSCs but rapidly downregulated transcriptionally upon activation retained H3K4me3 and gained H3K27me3 in ASCs (Figure 2D), suggesting that induction of H3K27me3 at TSSs may lead to rapid inhibition of QSC-specific genes upon SC activation. In addition, the level of H3K27me3 markedly increased in the gene body and intergenic regions during SC activation (Figure S3B). The dramatic increase in H3K27me3 upon SC activation correlated well with the transcriptional changes of the H3K27 methyltransferase Ezh2 and demethylase Jmjd3 (Figure S4B). SC activation was at least partly dependent on the upregulation of Ezh2 and the subsequent increase of H3K27me3 because knockdown of Ezh2 by siRNA inhibited the activation of SCs on myofibers in ex vivo cultures (Figure S4C).

Taken together, these data suggest that the chromatin of QSCs is maintained at a permissive state with a large number of genes marked by H3K4me3 and few marked by H3K27me3 at the TSSs. SC activation is accompanied by the retention of H3K4me3 and acquisition of H3K27me3. By accumulating H3K27me3 upon SC activation, the chromatin is converted to a more repressed state.

### Identification of Bivalent Domains in QSCs

**Figure 3:**
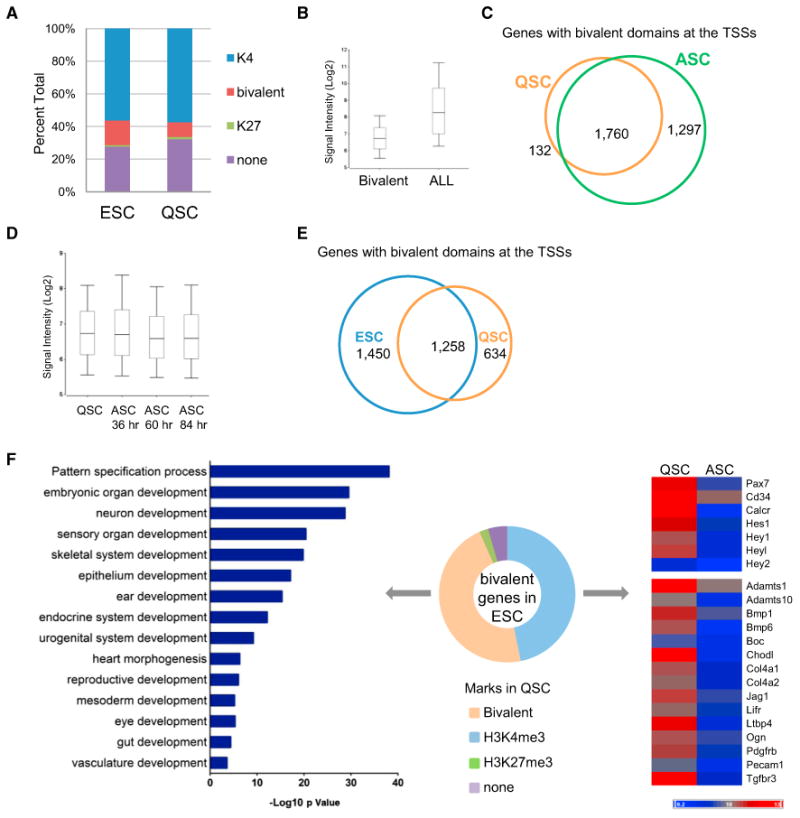
Identification of H3K4me3 and H3K27me3 Bivalent Chromatin Domains in QSCs (A) Comparison of the proportion of genes marked by one of the four H3K4me3 and H3K27me3 patterns, H3K4me3 only (K4), H3K27me3 only (K27), both (bivalent), and neither (none), in ESCs and QSCs. (B) Box and whisker plot of the expression level of genes that were found bivalent at the TSS in comparison to all genes in QSCs. (C) Venn diagram of genes with bivalent domains at the TSS in QSCs and ASCs. Among the 1,892 genes marked by bivalent domains in QSCs, 1,760 were also found in ASCs. (D) Box and whisker plot of the expression level of genes bivalent at the TSS in QSCs and ASCs of different time points in muscle regeneration. (E) Venn diagram of genes with bivalent domains at TSSs in ESCs and QSCs. (F) H3K4me3 and H3K27me3 patterns in QSCs of genes that are marked by bivalent domains in ESCs. The middle pie chart depicts the proportion of bivalent ESC genes that exhibited different H3K4me3 and H3K27me3 marks in QSCs. Among all genes that are bivalent in ESCs, 46% were found bivalent in QSCs (orange), and another 46% were found to be H3K4me3 only (blue). GO analysis of genes that were found bivalent in QSCs is shown by the bar graph on the left panel. The heatmap on the right panel depicts the expression level in QSCs and ASCs of representative genes that are bivalent at TSS in ESCs but H3K4me3 only in QSCs. The top panel of the heatmap shows genes known as QSC markers, and the bottom panel shows 15 of all 411 genes that encode glycoproteins (p = 10^−39^). See also Figure S5.

Bivalent chromatin domains composed of both H3K4me3 and H3K27me3 modifications mark a large number of key regulators of lineage differentiation in ESCs in culture (Bernstein et al., 2006; Mikkelsen et al., 2007; Marks et al., 2012). Analysis of our QSC ChIP-seq data revealed 1,892 bivalent domains at TSSs across the genome (Figure 3A). Consistent with their poised state of transcription, genes marked by bivalent domains at their TSSs in QSCs were either not transcribed or transcribed at very low levels (Figure 3B). Over 93% of these genes remained bivalent in ASCs and exhibited no change in their expression level (Figures 3C and 3D). We further compared the identity of genes with bivalent domains in ESCs and QSCs and found 1,258 common to both cell types, making up 46% of the total number of genes marked by bivalent domains in ESCs (Figure 3E). Surprisingly, the bivalent domains in QSCs marked a large number of nonmyogenic regulators of developmental processes of multiple organs and tissues (Figure 3F). Although SCs give rise to only myogenic progeny during adult muscle regeneration, it is possible that SCs retain the potential to adopt nonmyogenic fates given the poised nature of genes marked by bivalent domains.

Our comparison of bivalent domains in ESCs and QSCs also revealed that approximately 47% of the bivalent genes in ESCs were “H3K4me3 only” and expressed at high levels in QSCs (Figure 3F). This list of genes included a large number of QSC-specific genes as revealed by our microarray analysis, such as known markers of SCs, *Pax7, Cd34*, and Calcitonin receptor (*Calcr*) (Joe et al., 2010; Cheung et al., 2012), and Notch target genes *Hey1, Hey2*, and *Heyl*. Notably, this list of genes was dominated by genes encoding glycoproteins. Given that glycoproteins are integral membrane proteins that often play an important role in cell-cell and cell-matrix interactions (Moremen et al., 2012), these glycoproteins that expressed at high levels in QSCs may be important mediators of niche interaction.

**Figure 4:**
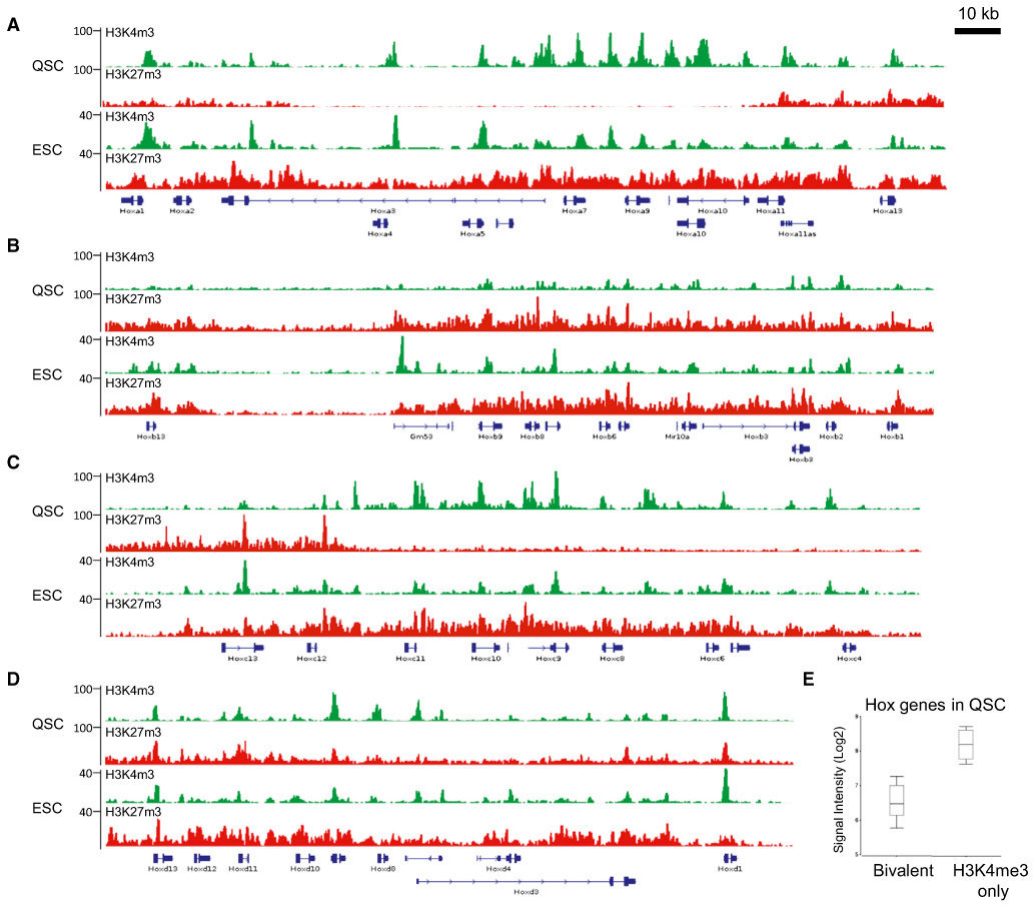
The *Hox* Loci Exhibit a Mosaic Pattern of Genes Marked by Bivalent Domains (A–D) The distribution of H3K4me3 and H3K27me3 at the four *Hox* loci in QSCs and ESCs. (E) Box and whisker plot of the expression levels of *Hox* genes that are bivalent at the TSSs and those that are marked by H3K4me3 only in QSCs. See also Table S3.

A large number (246) of transcription factors, many of which are well-documented regulators of morphogenesis of tissues other than skeletal muscle, were found to be bivalent at their TSS in QSCs (Table S3; Figure S5A). Nearly all of the bivalent transcription factors in QSCs are PcG targets in murine ESCs, including many *Hox* genes (Boyer et al., 2006). In ESCs, all four *Hox* loci are bound by the PcG complex and are bivalent; each cluster is marked by a broad H3K27me3 domain, and each gene within the cluster is marked by a sharp H3K4me3 peak at its TSS (Figures 4A–4D). Interestingly, in QSCs, the *Hoxb* and *Hoxd* loci exhibited similar bivalency to that in ESCs (Figures 4B and 4D), whereas the *Hoxa* and *Hoxc* loci exhibited a mosaic pattern of H3K4me3-only mark and bivalency. Although the genes at the two ends of the *Hoxa* locus were bivalent in QSCs, the ones at the center of this cluster, including *Hoxa3–Hoxa7, Hoxa9*, and *Hoxa10*, were marked by H3K4me3 only (Figure 4A). Similarly, bivalent *Hoxc* genes were present at the 5′ end of the locus, and H3K4me3-only ones were clustered at the 3′ end (Figure 4C). The H3K4me3-only *Hox* genes not only expressed at a much higher level than those that were bivalent in QSCs (Figure 4E), but they also generally decreased their expression following SC activation, providing evidence that they may play a role in the regulation of QSC function.

### H3K4me3 and H3K27me3 Patterns of Myogenic Genes in QSCs and ASCs

**Figure 5:**
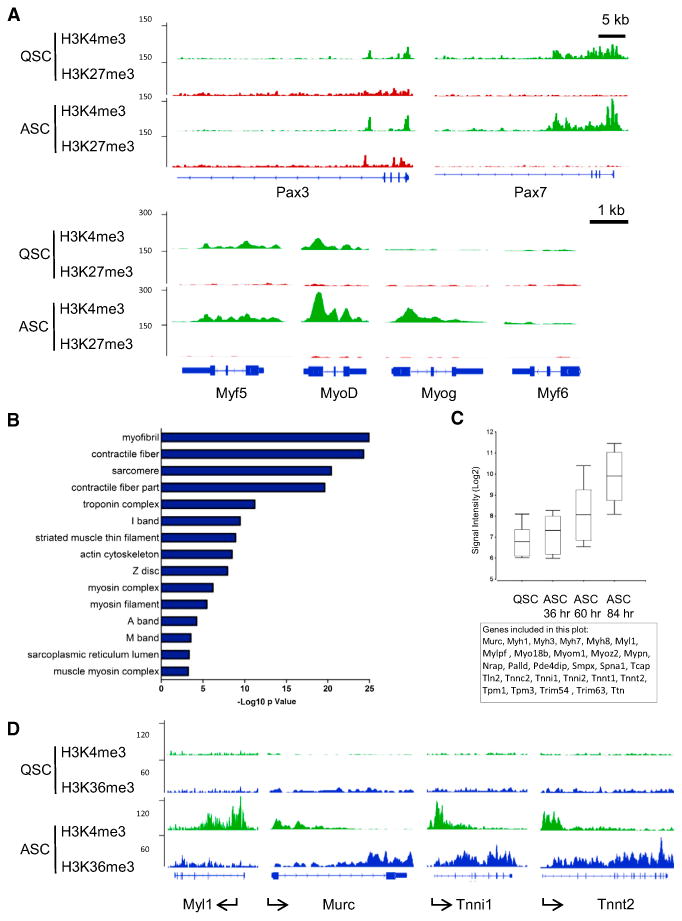
Chromatin Patterns of Myogenic Genes (A) The distribution of H3K4me3 and H3K27me3 at the TSSs of myogenic transcription factors Pax3, Pax7, Myf5, MyoD, Myogenin (Myog), and Myf6. (B) GO analysis of genes that were neither H3K4me3 nor H3K27me3 in QSCs but acquired H3K4me3 in ASCs. (C) Box and whisker plot of the expression levels of all genes associated with the GO terms listed in (B). (D) The distribution of H3K4me3 and H3K36me3 on representative genes associated with the GO terms listed in (B).

Myogenesis is regulated by a number of transcription factors, including Pax3, Pax7, Myf5, MyoD, Myogenin, and Myf6, in a temporal manner (Bentzinger et al., 2012). Interestingly, among these myogenic transcription factors, only Pax3 was found to be bivalent in QSCs (Figure 5A). This is consistent with the low expression level of Pax3 in limb SCs in adult mice. In contrast, Pax7 and Myf5, which are expressed at high levels in both QSCs, were marked only by H3K4me3 at their TSSs (Figure 5A). Despite the absence of detectable MyoD protein in QSCs (Figures S1B and S1E), we found the presence of MyoD transcript in QSCs, which did not exhibit significant change with activation (see Discussion). Consistent with the expression of MyoD transcript, H3K4me3 was also detected at its TSS in QSCs. Myogenin, which is expressed only in differentiating myoblasts, and Myf6, which exhibited no expression in QSCs or ASCs as revealed by our microarray analysis, were not marked by either H3K4me3 or H3K27me3 in QSCs (Figure 5A). Upon activation, among these six myogenic transcription factors, only *Myogenin* showed a significant change by gaining H3K4me3 at its TSS. *Pax3* remained bivalent, *Pax7, Myf5*, and *MyoD* all retained the H3K4me3 mark at their TSSs, and *Myf6*, whose expression was not induced in ASCs, remained unmarked by either of the histone marks. It will be intriguing to determine whether this temporal acquisition of H3K4me3 at the TSS of *Myogenin* underlies, or is merely a reflection of, its temporal expression pattern.

In addition to *Myogenin*, we have identified 177 genes that were not marked by either H3K4me3 or H3K27me3 at their TSSs in QSCs but were marked by H3K4me3 in ASCs. GO analysis revealed that these genes were highly enriched for genes encoding structural or functional proteins of the contractile properties of the muscle (Figure 5B). As expected from their function in the muscle, these genes were also among the most upregulated genes upon SC activation (Figure 5C). Reminiscent of the aforementioned high level of expression of MyoD in QSCs, the transcripts of a number of structural proteins, including some myosins and troponins that are not produced until after terminal differentiation and fusion of myoblasts, were significantly upregulated in ASCs 60 hr after injury. In addition to being marked by H3K4me3 at the TSSs in ASCs, each of these genes gained a broad domain of H3K4me3 that extended into the gene body and H3K36me3 that covered nearly the entire coding region (Figure 5D). Interestingly, these genes that acquired both H3K4me3 and H3K36me3 are also the most upregulated genes when MyoD is expressed in fibroblasts (Penn et al., 2004) and are bound by MyoD in differentiating myoblasts (Cao et al., 2010). These data suggest that MyoD cooperates with the Trithorax complex to induce the expression of at least a subset of its target genes.

### Increased H3K27me3 Level in QSCs with Age

Epigenetic integrity is an important element for maintaining normal stem cell function during aging (Liu and Rando, 2011; O'Sullivan and Karlseder, 2012). The appearance of aberrant stem cells with age has been demonstrated in mice in which the expression of some chromatin-modifying enzymes has been ablated in various stem cell compartments (Kamminga et al., 2006; Li et al., 2012). SCs exhibit a functional decline with age that results in impaired tissue regeneration upon acute muscle injury (Conboy et al., 2005; Brack et al., 2007). We therefore wanted to profile the histone modification patterns of SCs in aged animals and to determine whether the functional decline of SCs with age could be attributed to changes in the epigenome.

**Figure 6:**
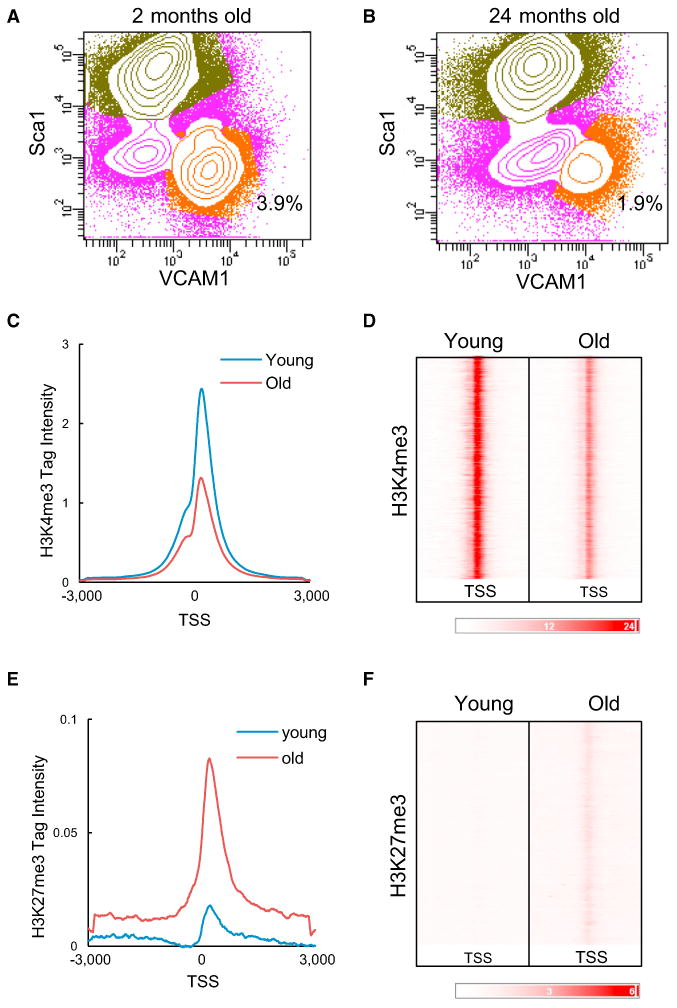
Changes in the H3K4me3 and H3K27me3 Profiles in QSCs with Age (A and B) FACS isolation of QSCs from hindlimb muscles of (A) 2-month-old and (B) 24-month-old mice. QSCs are shown in orange. The number indicates the percentage of QSCs among the total population of mononucleated cells in the muscle. (C) Distribution of H3K4me3 around the TSSs in young and old QSCs. Normalized tag intensity of H3K4me3 3 kb upstream and downstream of the TSSs across the genome is shown in the plot (p < 0.0001). (D) H3K4me3 intensity plot at TSSs in young and old QSCs. (E) Distribution of H3K27me3 around the TSSs in young and old QSCs. Normalized tag intensity of H3K27me3 3 kb upstream and downstream of the TSSs across the genome is shown in the plot (p < 0.0001). (F) H3K27me3 intensity plot at TSSs in young and old QSCs. See also Figure S6.

In order to compare the H3K4me3 and H3K27me3 epigenetic profiles between SCs in young and old mice, we isolated QSCs from 24-month-old mice to perform ChIP-seq analysis (Figure S6A). There was a decline in the percentage of QSCs in aged muscle compared to young muscle (Figures 6A and 6B), and consistent with our previous report (Conboy et al., 2005), QSCs from old mice required longer time to activate and reenter the cell cycle. Nevertheless, once activated, SCs isolated from old mice exhibited similar myogenic potential in ex vivo cultures under optimal differentiation conditions as those from young mice (Figure S6B). The genome-wide H3K4me3 profiles of QSCs from young and old mice were comparable. Both the total number of H3K4me3 peaks and their distribution relative to coding genes were highly similar (Figure S3B). Nearly all genes that were marked by H3K4me3 at their TSSs in QSCs from young mice retained the mark with age, despite a modest decline of the normalized tag intensity around TSSs (Figures 6C and 6D). Furthermore, the number of genes that were marked by H3K4me3 at their TSSs was nearly identical (12,062 in QSCs from young mice and 12,103 from aged mice).

In contrast to the overall low level of H3K27me3 across the QSC genome in young mice, this repressive chromatin mark appeared to accumulate and spread with age. In QSCs from young mice, 56% of the H3K27me3 peaks did not overlap with coding regions of known genes (Figure S3B). With age, additional H3K27me3 peaks were found in intergenic regions across the QSC genome, making up 67% of all the H3K27me3 peaks. These peaks were commonly between 0.5 and 1 kb in width and were located at various distances from the nearest coding gene (Figure S6C). The increase of H3K27me3 with age was also prominent at and around TSSs. There was a 4-fold increase in the H3K27me3 intensity near TSSs in QSCs from aged mice in comparison to those from young mice (Figure 6E), which resulted from both an increase of H3K27me3 intensity at the TSSs of genes that were already marked by this modification in QSCs from young mice and the acquisition of this mark with age on genes that were otherwise unmarked in QSCs from young mice (Figure 6F). Interestingly, over 30% of genes that acquired H3K27me3 at their TSSs with age were not expressed in either young or old QSCs (Table S3). For these genes, attaining the repressive H3K27me3 mark in old QSCs is unlikely to be a mechanism to suppress the transcription but, rather, may be a reflection of permanent loss of transcriptional potential with age.

### Histone Expression Is Affected by Gain of H3K27me3 in QSCs with Age

**Figure 7:**
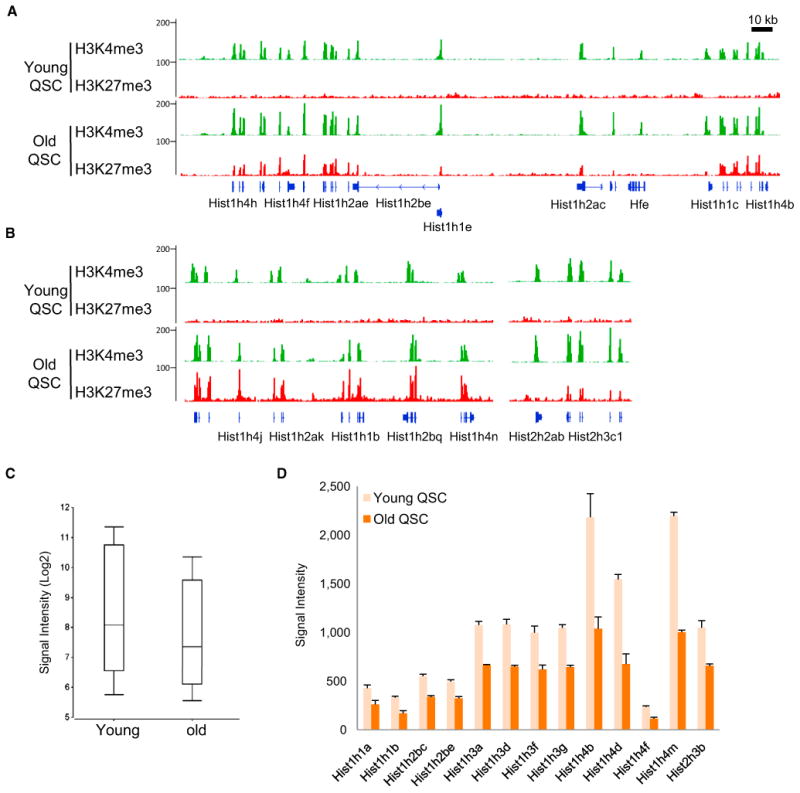
Histone Genes Acquire H3K27me3 in QSCs with Age and Exhibit a Reduced Level of Expression (A and B) Distribution of H3K4me3 and H3K27me3 in histone genes on (A) chromosome 3 and (B) chromosome 13 in young and old QSCs. (C) Box and whisker plot of the expression levels of all histone genes in QSCs from young and old mice. (D) The histone genes that exhibited a reduction in expression with age. Error bars represent SDs. See also Table S4.

We performed GO term analysis on the list of genes that gained the H3K27me3 mark at their TSSs in QSCs during aging. The terms “chromatin assembly” and “nucleosome,” which include a large number of histone genes, were enriched and of particular interest because a decrease in histone expression has been described in both yeast and cultured mammalian cells that have experienced replicative aging (Feser et al., 2010; O'Sullivan et al., 2010). In mouse, each core histone is encoded by 10–20 copies of histone genes that are clustered on chromosomes 3 and 13 (Wang et al., 1996a, 1996b). We examined the H3K4me3 and H3K27me3 enrichment patterns at these histone clusters and found that, whereas the histone genes were marked only by H3K4me3 in QSCs from young mice, they became bivalent with age (Figures 7A and 7B). The induction of H3K27me3 appeared to be highly specific to the histone genes because nonhistone genes within the clusters, such as the gene *Hfe* on chromosome 13, did not exhibit this change (Figure 7A).

Transcripts of the Histone genes are not polyadenylated at their 3′ ends, and the multiple copies of genes encoding the same histone share a high degree of homology in their coding regions (Marzluff et al., 2008). This poses a technical difficulty in using conventional RT-PCR analysis to evaluate the change of expression of each individual histone gene in QSCs with age. Therefore, in order to determine whether the acquisition of the H3K27me3 mark is associated with changes in the expression of histone genes, we performed gene expression microarray analysis with QSCs from 24-month-old mice and compared the data sets to those from young mice (Table S4). The comparison revealed an overall reduction in the expression of the histone genes that acquire H3K27me3 at their TSSs with age in QSCs (Figure 7C). Among the histone genes that are covered by the microarray analysis, 13 were found downregulated by more than 40% in old QSCs, including 2 histone 1 (H1) genes, 2 histone 2b (H2B) genes, 5 histone 3 (H3) genes, and 4 histone 4 (H4) genes (Figure 7D). Interestingly, despite the reduced expression with age in QSCs, the expression of these histone genes was induced to comparable levels by acute muscle injury in both young and old SCs (Figure S6E). Given that both cell-cycle-dependent and -independent regulation of histone expression have been described by Wang et al. (1996a, 1996b), our data suggest that SCs might utilize different mechanisms to regulate the expression of histone genes in the quiescent state and in the cell cycle. Although the decreased expression of these histones in QSCs is unlikely to be the causal event that leads to the delayed response to muscle injury in aged mice, it will be interesting to determine whether decreased histone expression affects SC homeostasis and thus contributes to the gradual loss of function of QSCs during aging.

## Discussion

The development of ChIP-seq technology in recent years has advanced our understanding of the epigenome of different types of cells (Mikkelsen et al., 2007). Using highly pure populations of QSCs and SCs activated in vivo, we have provided analysis of the kinetics of gene expression changes in SCs and their progeny in vivo during muscle regeneration. Furthermore, this report of gene expression profiles from young and old SCs provides a basis for studies of stem cell aging at the molecular level. In addition, we have successfully performed ChIP-seq analysis of QSCs and ASCs with regard to histone modifications to begin to understand the epigenetic features that define and regulate the quiescent stem cell state.

### Bivalent Domains in Adult Stem Cells

A surprising finding from our H3K4me3 and H3K27me3 ChIP-seq data from QSCs is the presence of a large number of genes that are marked by bivalent domains at their TSSs. Bivalent domains were first identified in ESCs at the *Hox* loci and later at the TSSs of a large number of genes that play important roles in specifying cell fate when ESCs are induced to differentiate in vitro (Bernstein et al., 2006; Mikkelsen et al., 2007). This report identifies prevalent bivalent domains in adult stem cells (Lien et al., 2011; Woodhouse et al., 2013), suggesting that the bivalent domains are a feature of not only ESCs in culture but also stem cells in their natural habitat in vivo. By comparing H3K4me3 and H3K27me3 profiles in ESCs and HF-SCs, Lien et al. identified merely 87 bivalent genes in HF-SCs (Lien et al., 2011). On the contrary, our analysis revealed more than 1,800 genes marked by bivalent domains at their TSSs (Figure 3B). It is even more striking that, similar to bivalent domains in ESCs, bivalent domains marked the TSSs of a large number of lineage-specific genes in QSCs (Figure 3F). Although sequential ChIP would be necessary to demonstrate definitively that both H3K4me3 and H3K27me3 marks are present at the TSSs of individual loci, the fact that such a high percentage of the same genes are enriched for both marks in QCSs and ESCs (Figure 3E) suggests that these TSSs truly are bivalent in the muscle stem cell lineage.

The bivalent domains in QSCs as an epigenetic memory may have implications in cellular reprogramming. The reprogramming of differentiated cells into induced pluripotent stem (iPS) cells has been achieved with a wide array of cell types (Takahashi and Yamanaka, 2006; Hanna et al., 2008). The reprogramming efficiency is typically extremely low with terminally differentiated cells and can be hundreds of times higher with stem or progenitor cells (Eminli et al., 2009). It has been recently demonstrated that the reprogramming efficiency of SCs isolated from mature adult muscles is more than 100 times that of lineage-committed myoblasts (Tan et al., 2011a). Because iPS reprogramming requires a stable reversion of the epigenetic state, the preservation of bivalent domains in QSCs and the similarity to ESCs may contribute to efficient reprogramming. Furthermore, it has been reported that early-passage iPS cells retain an “epigenetic” memory of their somatic cell of origin and preferably differentiate into the same type of cells (Polo et al., 2010). In contrast, iPS cells derived from muscle stem cells do not appear to preferentially differentiate along the myogenic lineage (Tan et al., 2011a). The permissive state of a large number of lineage genes marked by bivalent domains in QSCs may therefore also explain the unbiased differentiation potential of iPS cells generated from them (Taberlay et al., 2011; Tan et al., 2011a).

### Low H3K27me3 as a Chromatin Feature of Stem Cell Potential

Regardless of the difference in the number of genes marked by bivalent domains in QSCs and HF-SCs, one common chromatin feature between these two types of stem cells is the low level of the repressive H3K27me3 at the TSSs of annotated genes (Figure 2A; Lien et al., 2011). Only about 2,000 genes are marked by H3K27me3 in both SCs and HF-SCs. Particularly in QSCs, over 90% of the genes marked by H3K27me3 have bivalent TSSs. This leaves merely 127 genes that are “H3K27me3 only,” less than 0.5% of all the genes in the mouse genome (Waterston et al., 2002). Upon SC activation, the level of H3K27me3 increases significantly across the genome at both TSSs and intergenic regions.

Interestingly, a low level of H3K27me3 is also associated with the pluripotency of ESCs (Mikkelsen et al., 2007; Marks et al., 2012). A serum-independent culture condition has recently been established for ESCs (Marks et al., 2012). ESCs in this condition exhibit greater homogeneity in morphology and expression of pluripotent factors in comparison to those maintained with serum. Although the H3K4me3 pattern is similar in ESCs from these two culture conditions, a global redistribution of H3K27me3 occurs in ESCs in the absence of serum that results in a much-reduced level of H3K27me3 at promoters. In addition, the removal of H3K27me3 across the chromatin occurs during reprogramming of somatic cells into iPS cells, and cells that lack the expression of the H3K27me3 demethylase Utx fail to initiate reprogramming (Mansour et al., 2012). Therefore, the low level of H3K27me3 at promoters may be a common feature in both embryonic and adult stem cells. This general lack of repressive H3K27me3 and the presence of H3K4me3 at TSSs of a large number of genes may constitute a permissive chromatin state that underlies the potentiality of stem cells.

### Posttranscriptional Regulation of Adult Myogenesis

Increasing evidence has suggested that QSCs are not in a dormant state but, rather, primed for activation and differentiation (Cheung et al., 2012; Crist et al., 2012). As lineage-uncommitted progenitors residing in the muscle, QSCs respond to injury by rapidly activating the myogenic program. This primed state is reflected at the chromatin level by a general lack of the repressing H3K27me3 across the QSC genome and the presence of H3K4me3 at the TSSs of a large number of genes, including the myogenic regulatory factors, Myf5 and MyoD (Figure 5A), whose protein products are the primary activators of the myogenic program. MyoD protein is not detectable in QSCs but becomes highly induced by activation (Figures S1B and S1E).

Interestingly, unlike the MyoD protein, there are conflicting data as to the expression pattern of the MyoD transcript in QSCs and how the pattern changes with activation. The conclusion of an early single-cell PCR study was that the MyoD and Myf5 transcripts are expressed in a small subset of QSCs (Cornelison and Wold, 1997). However, in that study, a large percentage of ASCs were considered to be MyoD^−ve^, a finding that would be at odds with the observations that virtually 100% of ASCs are MyoD^+ve^ at the protein level. Therefore, a repeat of that study using a less-stringent cutoff for the determination of the expression of MyoD would likely reveal a much higher percentage of both QSCs and ASCs expressing MyoD at the transcript level. Using microarray analysis, Fukada et al. (2007) reported a significant induction of the MyoD transcript in ASCs from in vitro activation. By contrast, and similar to our data, Pallafacchina et al. (2010) detected MyoD transcripts in QSCs and found that levels of MyoD transcript did not exhibit a significant increase in ASCs during in vivo activation.

Similarly, whereas Myf5 was found to be expressed at high levels in QSCs by transcriptome analysis (Table S1), the protein product appeared to be at low abundance in QSCs and increase with activation (Figure S1E). Recently, it has been demonstrated that Myf5 transcript, together with microRNA-31 that regulates its translation, was sequestered in mRNP granules in QSCs (Crist et al., 2012). In ASCs, the mRNP granules were dissociated, and the level of microRNA-31 decreased, allowing the translation of Myf5 protein. In light of this study, it is possible that similar mechanisms are at play for the MyoD transcript and protein, whereby the protein expression is unequivocally a marker of ASCs, but the transcript is nevertheless expressed in QSCs. Thus, the transcription and translation of the MyoD gene may be uncoupled, and the level of MyoD protein may be regulated by similar posttranscriptional mechanisms as the Myf5 protein. Other posttranscriptional regulatory processes, such as splicing or RNA editing, could also be utilized by QSCs to allow the accumulation of the MyoD transcript without the production of the corresponding protein product. Because the specificity of transcription in different types of cells is determined by both the availability of transcription factors and the epigenetic features around their binding sites (Guccione et al., 2006), it is intriguing to consider the uncoupling of the transcription and translation of key myogenic regulatory factors as a feature of the primed state of QSCs.

Despite a lack of H3K27me3 on myogenic genes in both QSCs and ASCs, SC activation appeared to be accompanied by an increase in H3K27me3 in a large number of the bivalent TSSs of nonmyogenic lineage genes (Figures S5A and S5B), suggesting that these genes may be resolving to a fully repressed state. Our microarray analysis revealed a significant increase in the expression of the Polycomb component Ezh2 in ASCs (Figure S4B). Specific ablation of Ezh2 in myogenic progenitors during development leads to the transcriptional activation of nonmyogenic lineage genes, including ZIC-1, Isl1, and Tbx1 (Juan et al., 2011), all of which were marked by bivalent domains at their TSSs as revealed by our analysis. Ezh2-mediated H3K27me3 may therefore be critical to ensure the myogenic identity of SC progeny by suppressing the expression of genes that have the potential to drive alternative cell fates during adult muscle regeneration.

### Change of H3K27me3 with Age

Change of H3K27me3 level has been described in aged tissues in animals and *C. elegans*. A decrease in H3K27me3 level is associated with derepression of the *Ink4a/Arf* locus in pancreatic islets in old mice (Dhawan et al., 2009). H3K27me3 levels decrease strikingly with age throughout the tissues of *C. elegans* (Maures et al., 2011). Genetic manipulations of H3K27me3 methyltransferases or demethylases that increase the H3K27me3 level have been shown to extend the lifespan of worms and flies (Siebold et al., 2010; Maures et al., 2011). All of these data indicate that organismal aging is associated with a decrease of H3K27me3 in differentiated tissues.

Our ChIP-seq profiling of H3K27me3 revealed a global increase of H3K27me3 across the genome in SCs with age. This increase of H3K27me3 was prominent at both the TSSs and intergenic regions (Figures 6E, 6F, and S3B). Unlike the changes in the expression of Ezh2 and Jmjd3, which may contribute to the H3K27me3 increase upon SC activation in young animals, the expression of these genes did not exhibit a pattern of change that correlated with the accumulation of H3K27me3 in QSCs with age (Figure S6D). Whereas the vast majority of genes marked by H3K27me3 (including bivalent and H3K27me3 only) retained the mark with age in QSCs, the breadth of coverage was found to decrease at a subset of these genes, although the shifts were not associated with transcriptional changes. Redistribution of the Sir-silencing complex on the chromatin has been described in yeast cells during replicative aging (Kennedy et al., 1997). It is possible that a global increase of H3K27me3 leads to the redistribution of the PcG complexes across the genome in old QSCs, when the level of H3K27me3 methyltransferase and demethylase remains unchanged. The PcG complexes have been shown to be recruited to sites of DNA damage in mammalian cells (O'Hagan et al., 2008; Chou et al., 2010). Rossi et al. reported the presence of DNA double-strand breaks (DSBs) in hematopoietic stem cells with age (Rossi et al., 2007). Similarly, we have observed an accumulation of DSBs by γH2AX staining in QSCs from aged mice, whereas they are nearly undetectable in QSCs from young mice (G.W.C., unpublished data). Whether the accumulation of DSBs in old QSCs is a cause or consequence of the redistribution of the PcG complexes remains to be determined. Nevertheless, it is intriguing to consider that the increase of H3K27me3 with age may be the bridge between the reversible epigenetic changes and the irreversible genomic changes that occur in stem cells.

### Reduction in Histone Biosynthesis in Chronological Stem Cell Aging

Histone reduction has been observed in budding yeast and cultured mammalian cells during replicative aging (Feser et al., 2010; O'Sullivan et al., 2010). In late passages of human fibroblasts, a decrease in histone biosynthesis is caused by telomere shorting as a DNA damage response and appears to contribute to replicative senescence (O'Sullivan et al., 2010). Reduction in histone biosynthesis leads to transcriptional dysregulation in old yeast cells, and elevated expression of histones promotes the extension of replicative lifespan (Feser et al., 2010). These findings raise an interesting question as to whether a reduction in histone biosynthesis is a universal manifestation of cellular aging across eukaryotic species.

As a population of cells that lasts the lifetime of the organism, adult stem cells undergo both replicative aging when they divide and chronological aging when they remain in quiescence (Liu and Rando, 2011). QSCs provide an excellent model to study chronological aging due to their extremely low turnover. We report here that the transcription of histones decreases in QSCs during chronological aging (Figure 7C). In old QSCs, the reduction of histone expression is associated with acquisition of H3K27me3 at the histone loci (Figures 7A and 7B). This raises an intriguing question as to how the reduced level of histones affects cellular processes and function of nondividing cells.

A large body of biochemical and structural evidence has demonstrated that transcriptional elongation by RNA Pol II involves eviction and exchange of core histones in a histone chaperon-dependent manner (Workman, 2006). After the passage of RNA pol II, both evicted and newly synthesized histones can be incorporated into nucleosomes to restore chromatin configuration (Dion et al., 2007; Jamai et al., 2007). Histone turnover therefore accompanies active transcription in the absence of DNA replication. The nucleosome disassembly and reassembly in this process involve a number of factors, including recycling of evicted histones, synthesis of new histones, and availability of histone chaperones. In budding yeast, a moderate decrease in histone expression influences the DNA damage response, whereas substantial reduction of histones leads to a genome-wide decrease of nucleosome occupancy and global transcriptional noise (Celona et al., 2011). In response to a decrease in nucleosome occupancy, genes located within 20 kb from the telomeres are commonly derepressed in old yeast cells, whereas genes farther away from telomeres can be both up- and down-regulated. As such, reduced histone expression may have a broad impact on the global transcription in QSCs from old animals.

### Concluding Remarks

Functional decline of stem cells has been associated with many age-related conditions and diseases (Rossi et al., 2008). Because attenuation or reversal of the age-induced functional decline of stem cells has been achieved by modulating the environment to which they are exposed or their ability to cope with the aging environment (Conboy et al., 2005; Adler et al., 2007; Villeda et al., 2011), it has been postulated that the aging of stem cells occurs largely at the epigenetic level (Oberdoerffer et al., 2008; Liu and Rando, 2011; O'Sullivan and Karlseder, 2012; Rando and Chang, 2012). By profiling the histone methylation patterns of QSCs from young and aged mice, we provide here direct evidence for the epigenetic aspect of the aging of SCs. These findings not only reveal epigenetic features associated with the normal function of adult stem cells but also provide a framework for dissecting the epigenetic changes that impair stem cell function with age. Understanding the fundamental mechanisms of stem cell aging is necessary to develop interventions for age-related conditions and diseases that are related to the decline of stem cell function and, ultimately, to promote healthy aging.

## Experimental Procedures

All animal protocols were approved by the Administrative Panel on Laboratory Animal Care of the VA Palo Alto Health Care System. Additional details about the methods described below are provided in the Extended Experimental Procedures.

### Isolation of SCs

Hindlimb muscles were collected, finely minced, and subjected to Collagenase II and Dispase (Invitrogen) digest. The resulting suspension was then passed through a 20G needle to release the associated SCs. Cell suspensions were filtered through 45 mm cell strainers. The mononuclear cell suspension was then stained with a cocktail of antibodies and sorted on a BD FACS Aria II or BD FACSAria III cell sorter.

### Microarray Analysis of SCs

RNA isolation from SCs was performed with the TRIzol reagent (Invitrogen). The RNA was then processed and assayed by Affymetrix GeneChip Mouse Gene 1.0 ST Arrays.

### ChIP-Seq

FACS-sorted cells were immediately crosslinked with 1% formaldehyde following standard ChIP protocols. Each ChIP reaction was performed with 10^6^ cells and 5 μg of antibody. A library for deep sequencing was generated with Illumina ChIP-seq Sample Prep Kit with 15 cycles of PCR amplification.

## Supplementary Material

Supplemental table S1

Supplemental table S2

Supplemental table S3

Supplemental table S4

Supplemental text and figures
