# Supplementary material for: Chromatin Modifications as Determinants of Muscle Stem Cell Quiescence and Chronological Aging
Source: Cell Rep. Author manuscript; Available in PMC 2014 Jul 18. (PMC4103025; doi:10.1016/j.celrep.2013.05.043)
Supplement: Supplemental text and figures [file NIHMS588696-supplement-Supplemental_text_and_figures.pdf]

Cell PRESS Open ACCESS

EXTENDED EXPERIMENTAL PROCEDURES

Animals

Eight-week-old C57BL/6 and ROSA26$^{eYFP/eYFP}$ were purchased from Jackson Laboratory. Pax7$^{CreER/CreER}$ mice were kindly provided by Charles Keller at the Oregon Health and Science University. Twenty-four-month old C57BL/6 mice were purchased from the National Institute of Aging. Only male mice were used in all of the experiments. Tamoxifen injection for Cre recombinase activation and $BaCl_2$ injury to hindlimb muscles were performed as described previously (Cheung et al., 2012). Mice were housed and maintained in the Veterinary Medical Unit at Veterans Affairs Palo Alto Health Care Systems. Animal protocols were approved by the Administrative Panel on Laboratory Animal Care of the VA Palo Alto Health Care System.

Isolation of SCs

Hindlimb muscles were dissected from mice following euthanization and kept in wash solution (Ham's F10 media supplemented with 10% horse serum) until all of the muscles have been collected. The muscles were then finely minced with a surgical scalpel and digested in 2 mg/ml Collagenase II (Worthington, 340 U/mg) in the wash solution for 90 min with agitation. Dissociated fibers were washed with wash solution and further digested in 10 ml wash solution containing 100 U/ml Collagenase II and 2 U/ml Dispase (Invitrogen) for 30 min in shaking water bath. The resulting suspension was then passed through a 20-G needle with a 10-ml syringe 10 times to release the associated SCs. Cell suspensions were then wash two more times and filtered through a 45-μm cell strainer. Mononuclear cells from two mice were resuspended in 500 μl wash solution and stained with VCAM1-biotin, CD31-APC, CD45-APC and Sca1-Pacific-Blue antibodies at 1:100 dilution at 4°C with gentle agitation for 30 min. Cells were washed once and resuspended in 500 μl wash solution and stained with Streptavidin-PE-Cy7 at a 1:100 dilution at 4°C with gentle agitation for 15 min. Cells were then washed once with wash solution and sorted using a 70 μm nozzle on a BD FACS Aria II or BD FACSAria III cell sorter equipped with 488 nm, 633 nm and 405 nm lasers. From each sort, we plated 5,000 cells on Poly-D Lysine- and ECM- coated slides to assess the purity of the sorted population.

Microarray Analysis of SCs

RNA isolation from SCs was performed with the Trizol reagent (Invitrogen) following the manufacturer's instructions. The quality of the RNA was assessed with Agilent RNA 6000 Nano Kit on a 2100 Bioanalyzer. Processing of the RNA and hybridization to Affymetrix GeneChip Mouse Gene 1.0 ST Arrays were performed by the Protein and Nucleic Acid (PAN) Facility at Stanford University. Three replicates were used for each sample. Array data sets were analyzed with Cluster (Eisen et al., 1998) and/or Partek. Gene Ontology analysis was performed with DAVID (Huang da et al., 2009a, 2009b). Pathway analysis was performed with the Ingenuity software package.

ChIP-Seq

FACS-sorted cells were crosslinked with 1% formaldehyde at room temperature with gentle agitation for 10 min. The crosslink was terminated by Glycine at a final concentration of 0.125 M. Cells were then washed three times with ice-cold PBS and stored at −80°C until a sufficient number of cells had been obtained. ChIP was performed following standard ChIP protocols with the following major modifications: 10^6 cells were resuspended in 200 μl lysis buffer and sonicated to obtain DNA fragments from 150 to 500 bp in size. Each ChIP reaction was performed with 10^6 cells and 5 μg of antibody. The ChIP DNA was size-selected for fragments between 150-400 bp by 2% low-melt agarose gel electrophoresis (NuSieve). A library for deep sequencing was generated with Illumina ChIP-seq Sample Prep Kit with the following modification to the standard protocol: the adaptor solution was used at 1:50 dilution; 15 cycles of PCR amplification were performed immediately following adaptor ligation; Size-selection of fragments between 200-400 bp was performed after PCR amplification. ChIP-seq experiments were performed with two biological replicates. Sequencing was carried out using the Illumina/Solexa Genome Analyzer II system at Stanford Genome Technology Center. Reads were aligned to the mouse genome (mm9, build 37) by Bowtie (Langmead et al., 2009). Unique reads mapped to a single genomic location were used for peak detection by MACS with alignment of the ChIP input DNA as control (Zhang et al., 2008). The p value for peak calling was set at 10^{-5}. The subsequent analysis of the aligned reads and called peaks was performed with Homer (Heinz et al., 2010), BEDTools (Quinlan and Hall 2010), ChIPseeqer (Giannopoulou and Elemento 2011), GREAT (McLean et al., 2010) and Spotfire programs. The bivalent domains were identified by intersecting the H3K4me3 and H3K27me3 peak files with BEDTools with at least a 5 bp overlap.

Isolation, siRNA Transfection, and Immunofluorescence of Single Myofibers

The Extensor Digitorum Longus (EDL) muscles were carefully dissected from mice following euthanization and digested in 2 mg/ml Collagenase II prepared in wash solution (see above) at 37°C for 90 min with gentle agitation. The digested EDL muscles were then triturated with a wide bore glass pipet in 15 ml wash solution in a 100-mm tissue culture dish. Individual fibers were washed three times before they were fixed, cultured to allow SC activation, or transfected with siRNA. To fix fibers, fibers were incubated with 2% paraformaldehyde at room temperature for 5 min. To culture fibers ex vivo, about 100 fibers were maintained as suspension in 5 ml growth media in 60-mm dishes for 2-3 days. To transfect fibers with siRNA, we placed about 60 fibers in 2 ml media in a

35-mm dish. siRNA transfection was performed with Lipofectamine 2000 (Invitrogen) following manufacturer's instructions. 40 pmol of control or specific siRNA were used in each dish.

Antibodies

FACS antibodies: mouse CD31-APC (clone MEC 13.3, BD Bioscience or Biolegend), mouse CD45-APC (clone 30-F11, BD Bioscience or Biolegend), mouse Sca1-Pacific-Blue (clone D7, Biolegend), mouse VCAM1-biotin (clone 429, BD Bioscience or Biolegend), Streptavidin-PE-Cy7 (BD Biosciences or Biolegend). ChIP antibodies: H3K4me3 (Abcam 8898), H3K27me3 (Millipore 07449), H3K36me3 (Abcam 9050). Immunofluorescence antibodies: mouse Pax7 (Developmental Studies Hybridoma Bank), mouse MyoD (Clone 5.8A, Dako), mouse Myogenin (Clone F5D, BD Bioscience), GFP (Invitrogen A11122).

SUPPLEMENTAL REFERENCES

Eisen, M.B., Spellman, P.T., Brown, P.O., and Botstein, D. (1998). Cluster analysis and display of genome-wide expression patterns. Proc. Natl. Acad. Sci. USA *95*, 14863–14868.

Giannopoulou, E.G., and Elemento, O. (2011). An integrated ChIP-seq analysis platform with customizable workflows. BMC Bioinformatics *12*, 277.

Heinz, S., Benner, C., Spann, N., Bertolino, E., Lin, Y.C., Laslo, P., Cheng, J.X., Murre, C., Singh, H., and Glass, C.K. (2010). Simple combinations of lineage-determining transcription factors prime cis-regulatory elements required for macrophage and B cell identities. Mol. Cell *38*, 576–589.

Huang da, W., Sherman, B.T., and Lempicki, R.A. (2009a). Bioinformatics enrichment tools: paths toward the comprehensive functional analysis of large gene lists. Nucleic Acids Res. *37*, 1–13.

Huang da, W., Sherman, B.T., and Lempicki, R.A. (2009b). Systematic and integrative analysis of large gene lists using DAVID bioinformatics resources. Nat. Protoc. *4*, 44–57.

Langmead, B., Trapnell, C., Pop, M., and Salzberg, S.L. (2009). Ultrafast and memory-efficient alignment of short DNA sequences to the human genome. Genome Biol. *10*, R25.

McLean, C.Y., Bristor, D., Hiller, M., Clarke, S.L., Schaar, B.T., Lowe, C.B., Wenger, A.M., and Bejerano, G. (2010). GREAT improves functional interpretation of cis-regulatory regions. Nat. Biotechnol. *28*, 495–501.

Quinlan, A.R., and Hall, I.M. (2010). BEDTools: a flexible suite of utilities for comparing genomic features. Bioinformatics *26*, 841–842.

Zhang, Y., Liu, T., Meyer, C.A., Eeckhoute, J., Johnson, D.S., Bernstein, B.E., Nusbaum, C., Myers, R.M., Brown, M., Li, W., and Liu, X.S. (2008). Model-based analysis of ChIP-Seq (MACS). Genome Biol. *9*, R137.

Cell PRESS Open ACCESS

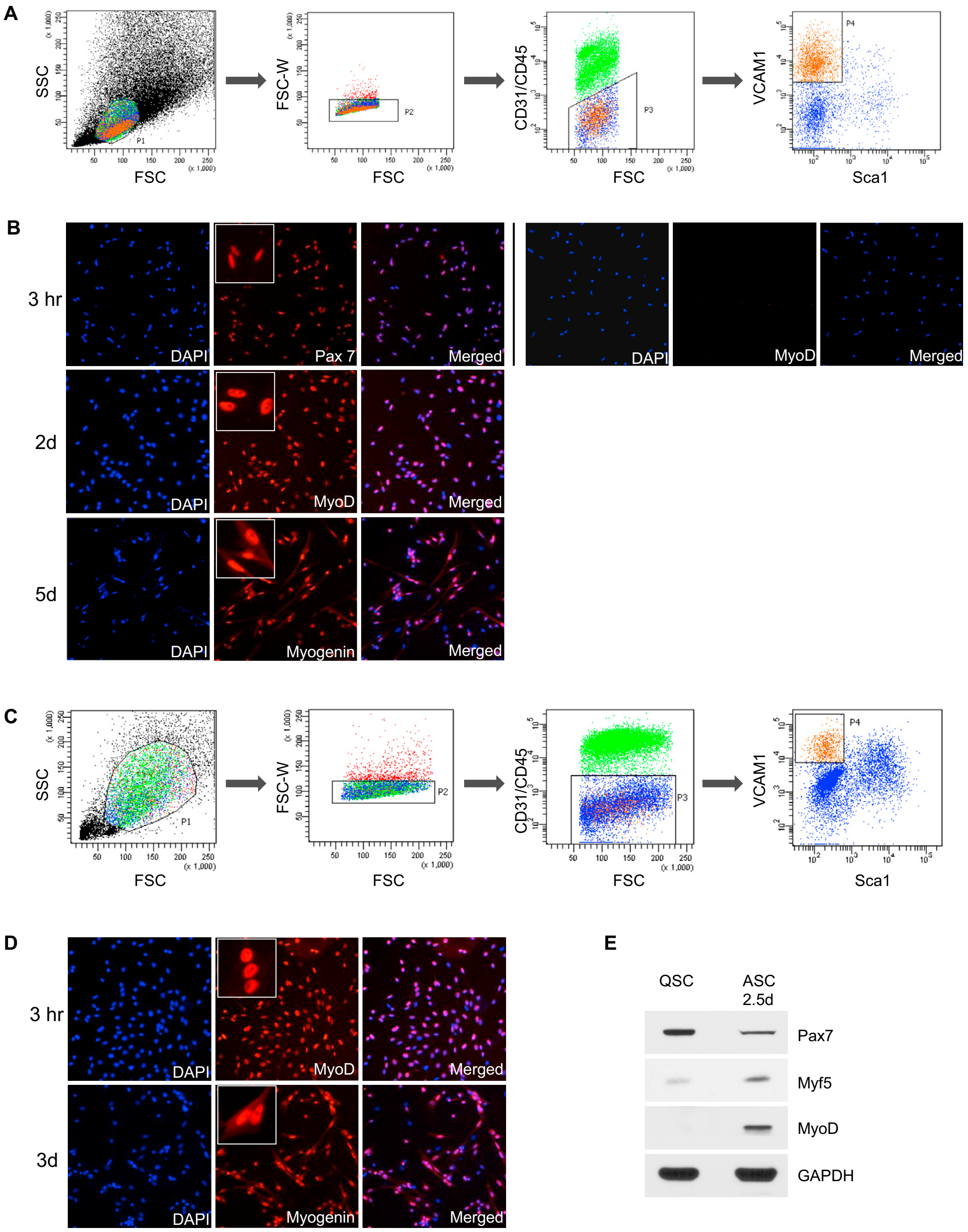

Figure S1. FACS Isolation of VCAM1$^+$ SCs from Adult Mice, Related to Figure 1

(A) Typical FACS plots of QSC isolation from young mice.

(B) Immunofluorescence of QSCs isolated from uninjured mice for specific myogenic markers. QSCs were plated after FACS isolation on ECM-coated surface. Cells were fixed 3 hr, 2 days and 5 days after plating and stained with Pax7 (left column) and MyoD (right column), MyoD and Myogenin antibodies, respectively.

(C) Typical FACS plots of ASC isolation from young mice 1.5 to 3.5 days after muscle injury.

(D) Immunofluorescence of ASCs isolated from mice 2.5 days after injury for specific myogenic markers. ASCs were plated after FACS isolation on ECM-coated surface. Cells were fixed 3 hr and 3 days after plating and stained with MyoD and Myogenin antibodies, respectively.

(E) Pax7, Myf5 and MyoD protein levels in QSCs and ASCs. Total protein was extracted from 10^5 QSCs and 10^5 ASCs isolated from mice 2.5 days after $BaCl_2$ injury and analyzed by SDS-PAGE and western blotting.

Cell PRESS
Open ACCESS

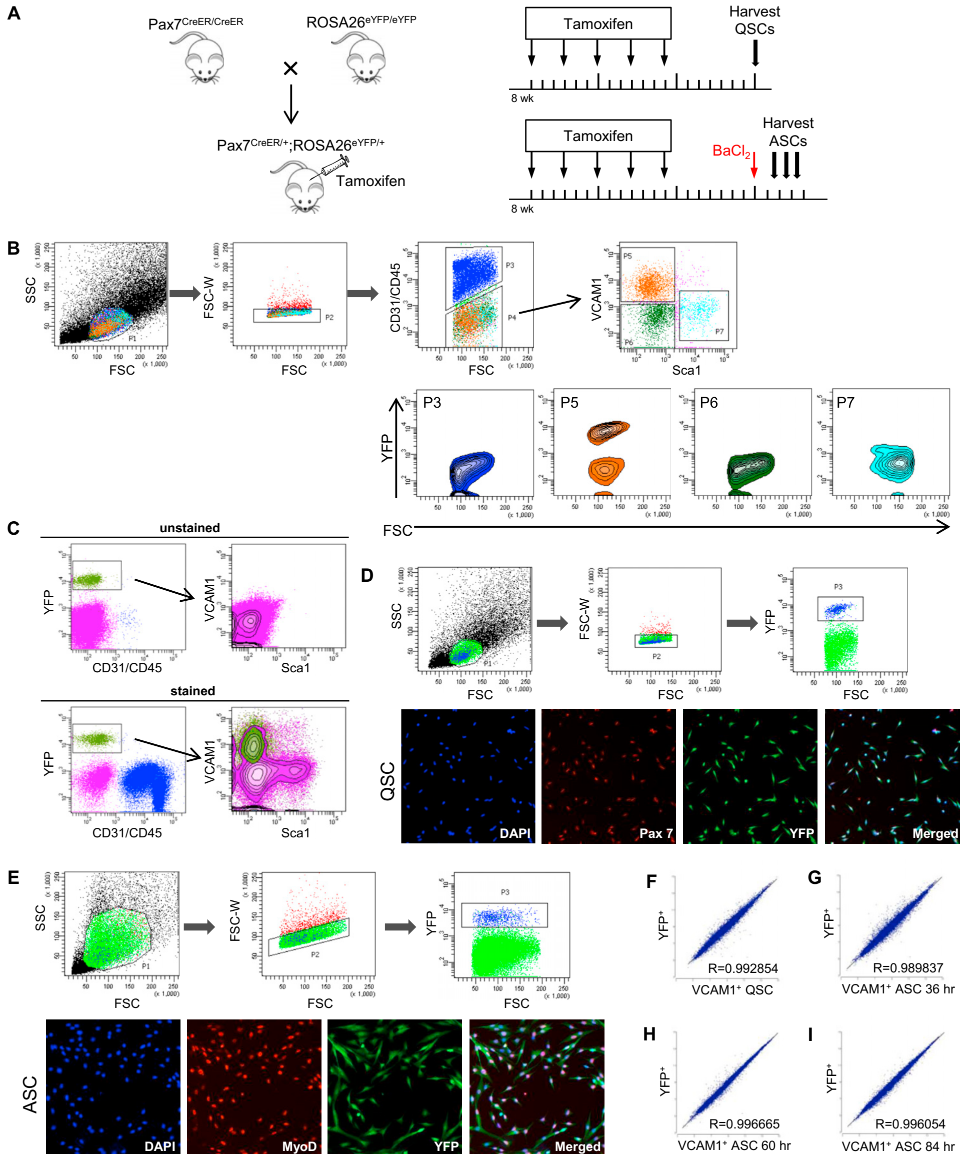

Figure S2. Validation of VCAM1 as a Positive Marker for SC Isolation, Related to Figure 1

(A) Transgenic mouse models to genetically label QSCs with YFP (left) and scheme for Tamoxifen injection, $BaCl_2$ injury when applicable, and cell harvest (right).

(B) FACS plots demonstrating that YFP^+ cells are found in the $VCAM1^+/CD31^-/CD45^-/Sca1^-$ population in resting muscles of the $Pax7^{CreER/+};ROSA26^{eYFP/+}$ mice only when injected with Tamoxifen. Top panels: Gates set for the $CD31^+/CD45^+$ (P3), $VCAM1^+/CD31^-/CD45^-/Sca1^-$ (P5), $VCAM1^-/CD31^-/CD45^-/Sca1^-$ (P6) and $Sca1^+/CD31^-/CD45^-$ (P7) populations. Bottom panels: FACS analysis of YFP expression of the above populations.

(C) FACS plots demonstrating all YFP-positive cells of the $Pax7^{CreER/+};ROSA26^{eYFP/+}$ mice injected with Tamoxifen are positive for VCAM1 and negative for CD31, CD45 and Sca1. Top panels: FACS profiles without antibody staining. Bottom panel: FACS profiles with antibody staining for VCAM1, CD31/CD45 and Sca1.

(D) Validation of Pax7 expression in YFP-positive cells of the $Pax7^{CreER/+};ROSA26^{eYFP/+}$ mice injected with Tamoxifen. Top panels: typical FACS plots of isolation of YFP-positive cells from uninjured mice. Bottom panels: immunofluorescence of YFP-positive cells fixed 12 hr after isolation with the Pax7 antibody from 2.5-day injured mice.

(E) Validation of MyoD expression in YFP-positive cells of the $Pax7^{CreER/+};ROSA26^{eYFP/+}$ mice injected with Tamoxifen followed by $BaCl_2$ injury. Top panels: typical FACS plots of isolation of YFP-positive cells from injured mice. Bottom panels: immunofluorescence of YFP-positive cells fixed 24 hr after isolation with the MyoD antibody.

(F–I) Scatter plots of the gene expression profiles of the $VCAM1^+/CD31^-/CD45^-/Sca1^-$ cells from wild-type mice and the YFP-positive cells from $Pax7^{CreER/+};ROSA26^{eYFP/+}$ mice injected with Tamoxifen.

Cell PRESS
Open ACCESS

A

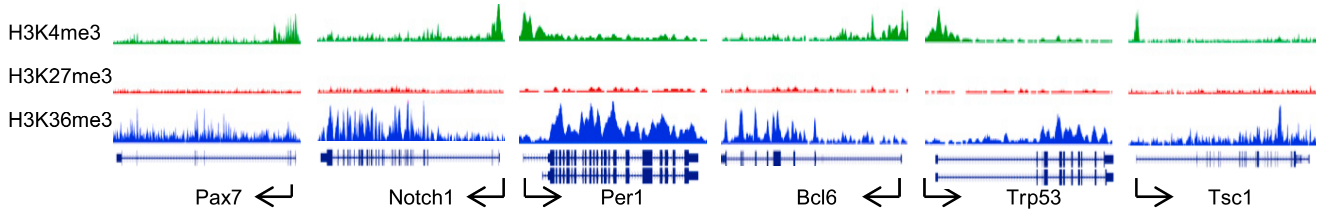

B

	H3K4me3			H3K27me3		
	young QSC	young ASC	old QSC	young QSC	young ASC	old QSC
Upstream of a gene	5,174	7,328	5,619	2,584	22,048	9,693
overlap gene start	12,062	12,076	12,103	1,550	3,342	2,271
inside gene	3,717	7,567	4,069	1,400	11,683	5,398
overlap gene end	372	406	343	226	527	217
downstream of a gene	3,151	4,070	3,460	1,391	11,580	6,018

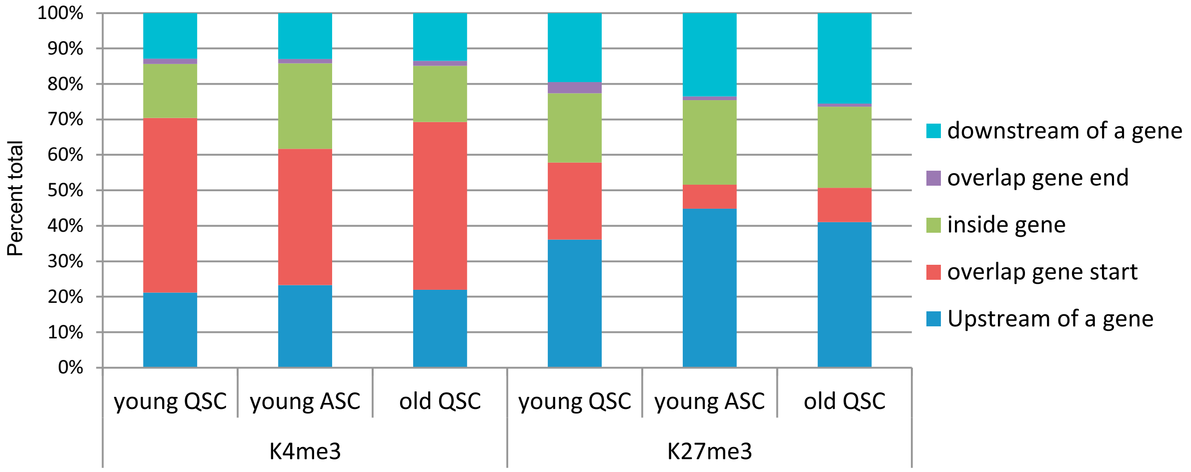

Figure S3. The Global Profile of H3K4me3 and H3K27me3 in QSCs and ASCs, Related to Figure 2

(A) Distribution of H3K4me3, H3K27me3 and H3K36me3 at the TSSs of representative genes that were highly expressed in QSCs.

(B) The number of peaks called for each ChIP-seq sample and their relative position to the closest annotated genes.

Cell PRESS
Open ACCESS

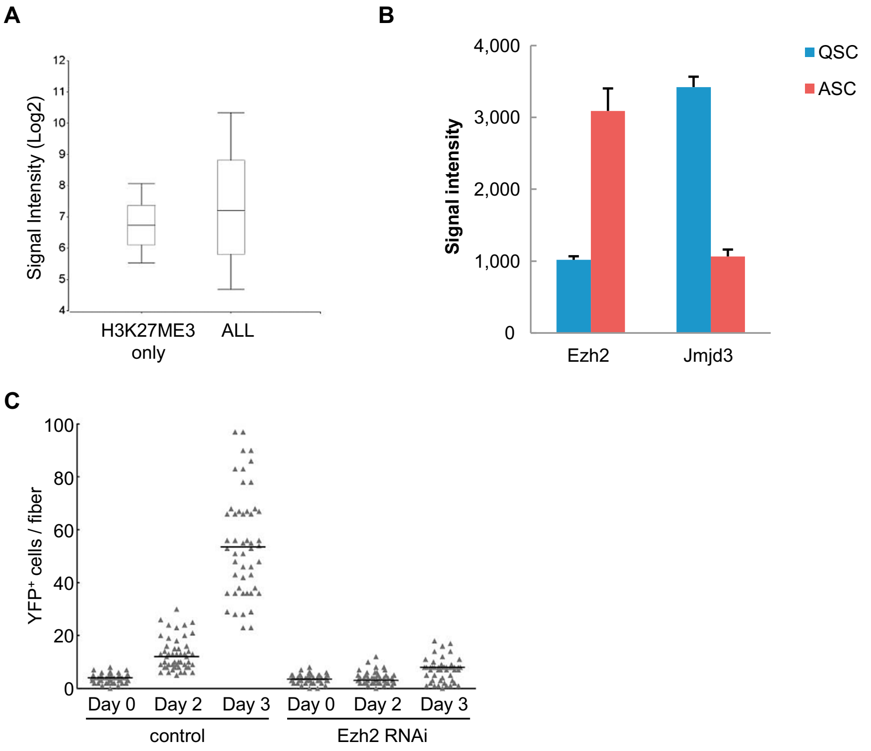

Figure S4. The Induction of H3K27me3 during SC Activation Is Associated with an Increase in Ezh2 Expression, Related to Figure 2

(A) Box and whisker plot of the expression levels of genes marked by H3K27me3 at their TSSs and of all genes in QSCs.

(B) Expression levels of Ezh2 and Jmjd3 in QSCs and ASCs from young mice. Error bars represent SDs.

(C) Freshly isolated myofibers from Pax7$^{CreER/+}$; ROSA26$^{eYFP/+}$ mice were transfected with siRNA targeting Ezh2 or a control siRNA immediately following isolation. Transfected fibers were cultured in SC growth media for up to 3 days. The numbers of YFP^{+ve} cells (SCs or their progeny) per fiber were quantified each day.

Cell PRESS
Open ACCESS

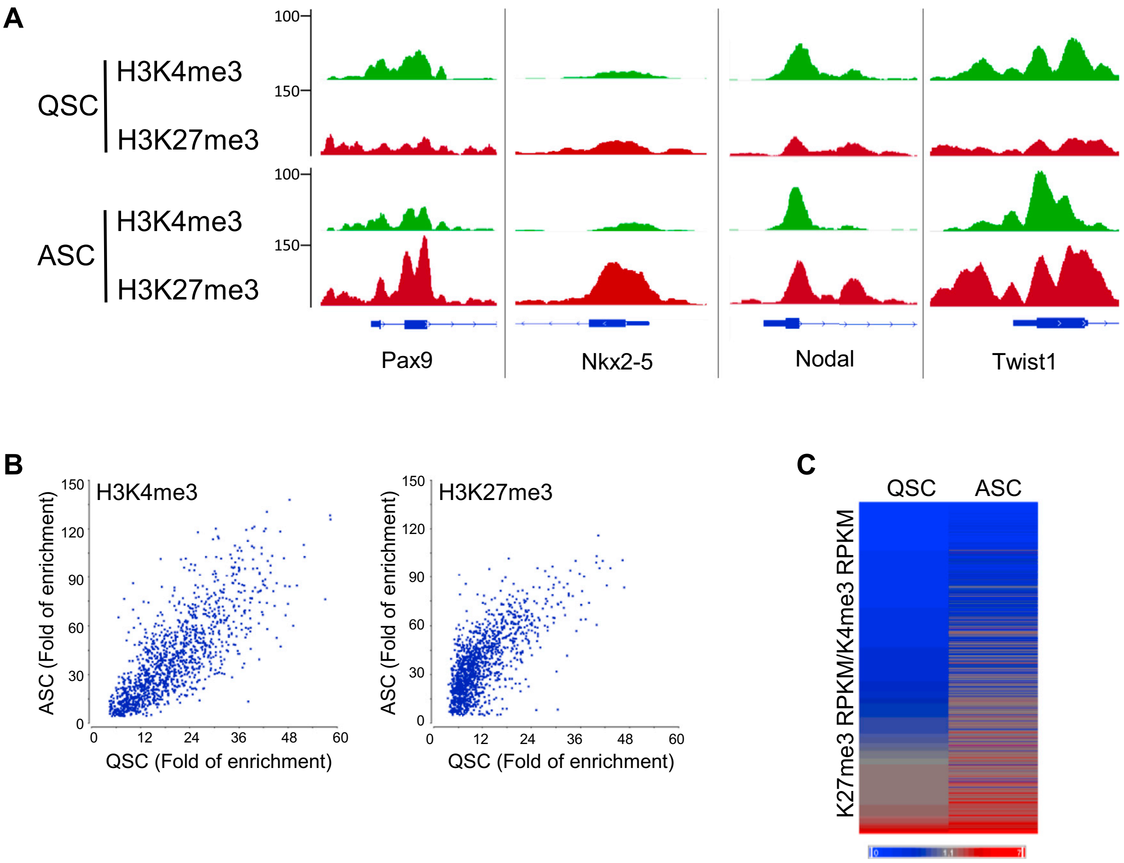

Figure S5. Changes of Bivalent Domains with SC Activation, Related to Figure 3

(A) Comparison of the H3K4me3 and H3K27me3 distribution at the TSSs of representative genes that were bivalent in QSCs and ASCs. The same scale is set for both populations.

(B) In order to determine whether the bivalent domains dynamically change with SC activation, we calculated the fold-enrichment of the H3K4me3 and H3K27me3 peaks separately at each bivalent domain in QSCs and ASCs. A scatter plot of the fold-enrichment values was generated for each modification between the two types of cells. Comparison of the two scatter plots representing H3K4me3 and H3K27me3 separately revealed while the fold-enrichment of most of the H3K4me3 peaks remained largely unchanged with SC activation, the fold-enrichment of the H3K27me3 peaks generally increased.

(C) The ratio of fold-enrichment of H3K27me3 over H3K4me3 of each QSC-specific bivalent gene was calculated in QSCs (left) and ASCs (right), respectively. The heat map was sorted by the ratio in QSCs from the smallest to the largest. The heat map clearly indicated that, consistent with the global increase of H3K27me3 in ASCs, the bivalent domains exhibited a general H3K27me3 increase upon SC activation.

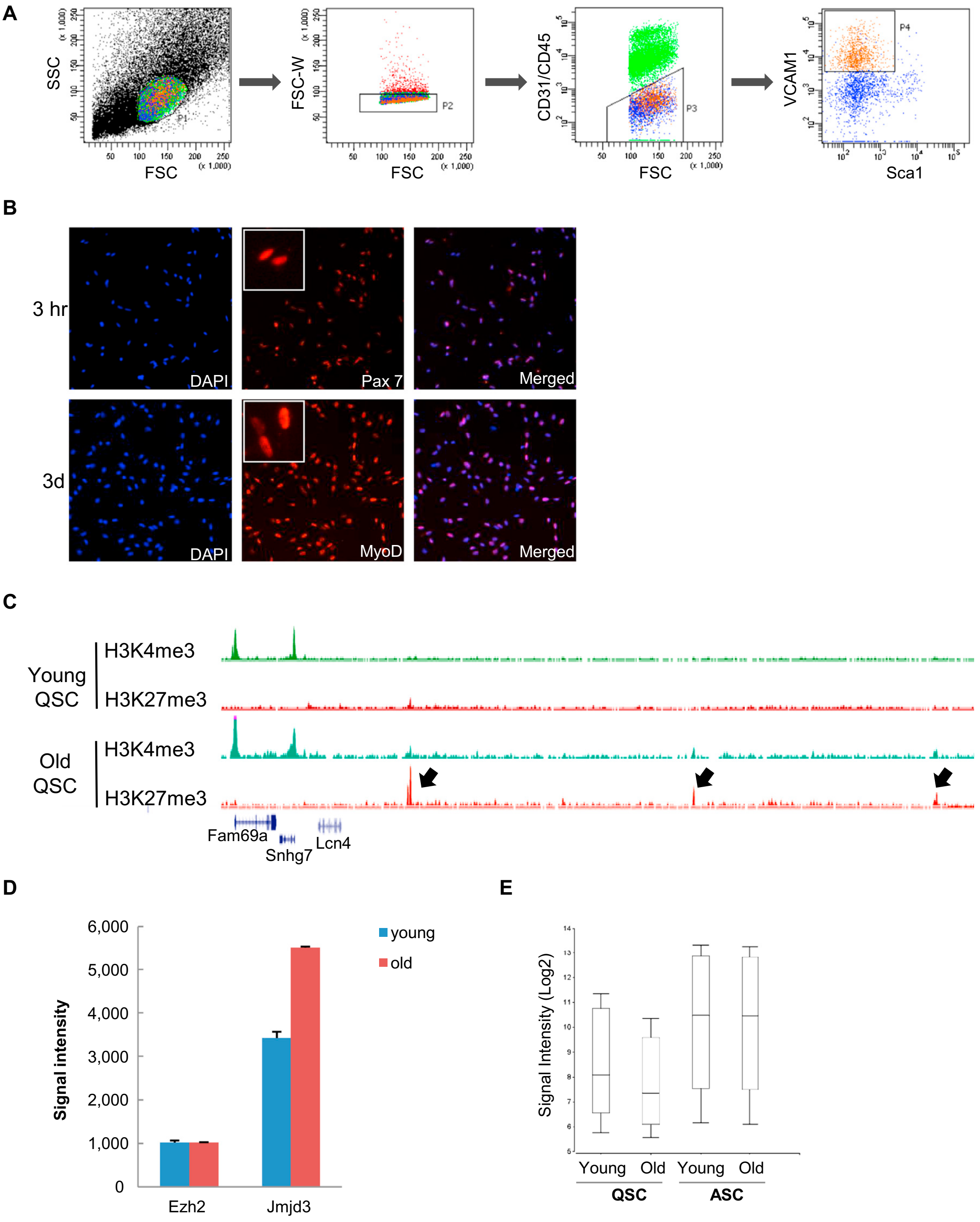

Figure S6. Isolation and Characterization of QSCs and ASCs from Aged Mice, Related to Figure 6

(A) Typical FACS plots of QSCs isolation from 24-month-old mice.

(B) Immunofluorescence of QSCs isolated from 24-month-old mice for specific myogenic markers. Cells were plated after FACS isolation on ECM-coated surface, and fixed 3 hr and 3 days after plating and stained with Pax7 and MyoD antibodies, respectively.

(C) Small H3K27me3 peaks detected in an intergenic region on the chromatin in old QSCs.

(D) Expression levels of Ezh2 and Jmjd3 in QSCs from young and old mice. Error bars represent SDs.

(E) Box and whisker plot of the expression levels of histone genes in QSCs and ASCs from young and old mice.
